# Single-Cell RNA-Seq Analysis Uncovers Distinct Functional Human NKT Cell Sub-Populations in Peripheral Blood

**DOI:** 10.3389/fcell.2020.00384

**Published:** 2020-05-26

**Authors:** Li Zhou, Indra Adrianto, Jie Wang, Xiaojun Wu, Indrani Datta, Qing-Sheng Mi

**Affiliations:** ^1^Center for Cutaneous Biology and Immunology Research, Department of Dermatology, Henry Ford Health System, Detroit, MI, United States; ^2^Immunology Research Program, Henry Ford Cancer Institute, Henry Ford Health System, Detroit, MI, United States; ^3^Center for Bioinformatics, Department of Public Health Sciences, Henry Ford Health System, Detroit, MI, United States

**Keywords:** natural killer T cells, single cell RNA sequencing, peripheral blood mononucleotide cells, gene express profile, cell population analysis, cell survival and proliferation

## Abstract

Vα24-invariant human natural killer T (NKT) cells comprise a unique subset of CD1d-restricted T cells with potent immune regulatory function and are involved in the development of a variety of human diseases. However, the lack of comprehensive molecular subset identities limits their objective classification and clinical application. Using unbiased single-cell RNA sequencing (scRNA-seq) of over 4000 unstimulated and 7000 stimulated human peripheral blood NKT cells, we identified four and five clusters of NKT cells from each NKT group, respectively. Our study uncovers multiple previously unrecognized NKT subsets with potential functional specificities, including a cluster of NKT cells with regulatory T cell property. Flow cytometry and Ingenuity Pathway Analysis confirmed the existence of these NKT populations and indicated the related functional capacities. Our study provides the unbiased and more comprehensive molecular identities of human NKT subsets, which will eventually lead the way to tailored therapies targeting selected NKT subsets.

## Introduction

Natural killer T (NKT) cells constitute a unique but heterogeneous subset of immune cells that arise from CD4^+^CD8^+^ cortical thymocytes, and feature characteristics of both conventional T and natural killer (NK) cells. NKT cells are potent immune regulators and skew immune responses toward either inflammation or tolerance through quickly secreting either T helper (Th) 1-, Th2-, Th17- or regulatory T-associated cytokines upon activation. Although the thymic development and functional differentiation of NKT cells is intensively studied in mice, it remains elusive how many human NKT subtypes exist, how they are functionally related to each other and how they differ from well-defined mouse NKT functional subpopulations. Several previous studies have shown that human NKT cells constitutively express various T cell markers such as TCR signaling complex CD3, and costimulatory receptors such as CD4, CD8, and CD28 (Gumperz et al., 2002; Lee et al., 2002; Montoya et al., 2007; Brennan et al., 2013). Like mouse NKT cells, human NKT cells can be divided into different functional subsets based on cytokine secretion specificities in different experiments and various conditions, such as Th1-like, Th2-like, Th17-like and Treg-like NKT cells (Lee et al., 2002; Moreira-Teixeira et al., 2012; Brennan et al., 2013). However, the classification of human NKT cells is likely to be biased by the limited information on the molecular bases for the differentiation and function of NKT cell subsets. Such biases, in turn, would limit the capability of the objective evaluation, classification and clinical application of human NKT cells.

To overcome some of the limitations and further explore the human NKT functional sub-lineages and related molecular basis, we used single-cell RNA sequencing (scRNA-seq) to assess the diversity of peripheral blood NKT cells at steady and activation states. The results of this study lead to a more objective classification of human NKT cells, uncover previously unrecognized NKT subsets, and pinpoint the potential molecular mechanisms of functional specificities of different NKT subsets. Overall, our analysis provides an unbiased and more comprehensive map of human peripheral blood NKT cells.

## Materials and Methods

### Human Peripheral Blood NKT Cell Enrichment, Sorting and Flow Cytometry

The study was performed in accordance with the Declaration of Helsinki, and the protocol was approved by the institutional review board at Henry Ford Health System. All subjects gave their informed consent for inclusion before they participated in the study. Healthy donors were recruited from Henry Ford Hospital. All healthy donors were non-smokers, had a normal BMI and normal blood pressure and were between 25 and 40 years of age.

**FIGURE 1:**
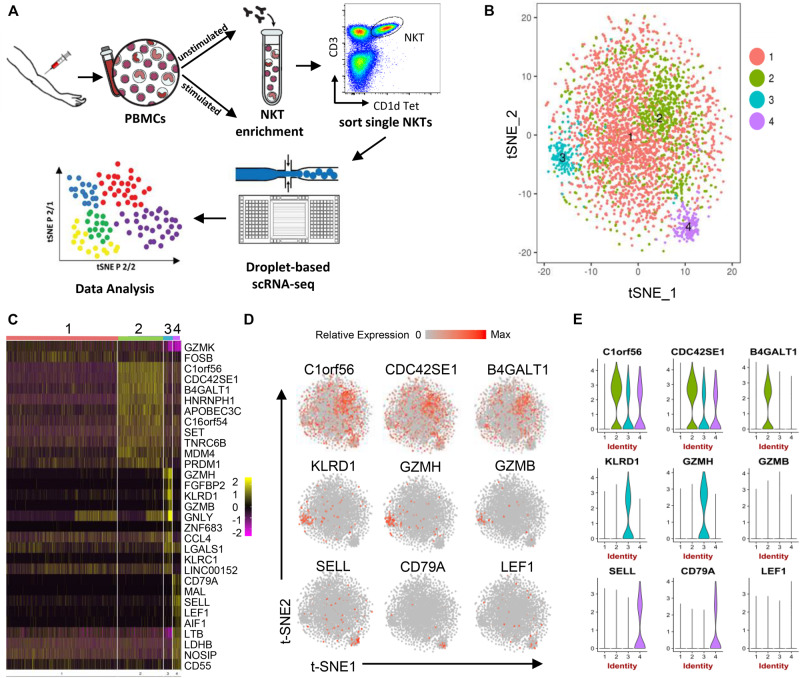
Human peripheral blood NKT cell heterogeneity at steady state delineated by single-cell RNA sequencing. **(A)** Workflow of experimental strategy: (i) isolation of human PBMCs from peripheral blood, followed by PMA/Ionomycin or vehicle treatment; (ii) NKT cell enrichment by autoMACS following CD1d-Tetrama-PE and anti-PE microbeads incubation; (iii) sorting single NKT cells; (iv) single-cell transcriptome profiling. **(B)** T-distributed stochastic neighbor embedding (t-SNE) plots generated by pooling of the three individual scRNA-seq libraries. Data sets were combined and aligned through the use of R package Seurat’s canonical correlation analysis (CCA) function. t-SNE dimensionality reduction analysis identified four major clusters. **(C)** Heatmap of the most differentially expressed genes in each cluster from **(B)**. **(D)** Feature t-SNE plots depicting the cluster-specific expression of top cluster-defining genes. **(E)** Violin plots illustrating the expression distribution of top cluster-defining genes across different clusters.

Peripheral blood mononuclear cells (PBMCs) from three healthy individuals were isolated from fresh blood within 2 h of collection using Ficoll-Plaque density gradient (Zhou et al., 2012). Single cell suspensions were stained with PE-hCD1d PBS-57 (NIH Tetramer Core Facility), followed by anti-PE microbeads (Miltenyi Biotec), and cells were then separated using autoMACS (Miltenyi Biotec). Separated NKT-enriched PBMCs were stained with anti-hCD3 and hCD1d PBS-57 antibodies and NKT cells (CD3^+^CD1d PBS57^+^) were then sorted on FACS Aria II (BD Bioscience) (Figure 1A).

For stimulated NKT cells, freshly isolated PBMCs were first stimulated with PMA/Ionomycin for 2 h, followed by NKT cell enrichment and sorting. For regular flow cytometry analysis, freshly isolated PBMCs were stained with different panels of antibodies, and flow cytometry were performed on FACSCelesta (BD Biosciences), and data was analyzed using FlowJo10.2.

### Single-Cell RNA Library Generation and RNA Sequencing

Single cell sequencing libraries were generated using the 10× Genomics Chromium Single Cell 3′ Reagent Kit (v2 Chemistry) and Chromium Single Cell Controller as previously described (Zheng et al., 2017). Briefly, about 5,000 cells were loaded into each reaction for gel bead-in-emulsion (GEM) generation and cell barcoding. Reverse transcription of the GEM (GEM-RT) was performed in a Thermocycler (Veriti^TM^ 96-Well Fast Thermal Cycler, Applied Biosynthesis; 53°C 45 min, 85°C 5 min, 4°C hold). cDNA amplification was performed after GEM-RT cleanup with Dynabeads MyOne Silane (Thermo Fisher Scientific) with the same Thermocycler (98°C 3 min; 98°C 15 s, 67°C 20 s, 72°C 1 min, repeat 12 cycles; 72°C 1 min, 4°C hold). Amplified cDNA was cleaned up with SPRIselect Reagent Kit (Beckman Coulter) followed by library construction procedure, including fragmentation, end repaired, adaptor ligation, and library amplification. A Bioanalyzer (Agilent) was used for library quality control. cDNA libraries were sequenced on an Illumina HiSeq 4000 using paired-end flow cells (Read 1, 26 cycles, i7 index 8 cycles; Read 2: 110 cycles) by the DNA Sequencing Core facility, University of Michigan.

### Single-Cell RNA Sequencing Data Analysis

Sequenced reads from scRNA-seq libraries were demultiplexed, aligned to the refdata-cellranger-hg19-1.2.0, barcode processed, and UMI counted using the 10X Genomics Cell Ranger (v2.0.1) pipeline (Zheng et al., 2017). 14,119 cells with 1,850 UMI counts/cell in average were selected via Cell Ranger for further analysis for all six samples.

Quality control metrics employed are as follows. Two strategies were employed to identify potential doublets: (1) cells expressing both XIST and Y chromosome genes; (2) cells expressing uncharacteristically high numbers of genes (>1,250). Low quality cells were excluded based on a low number of genes detected (<300) and/or having high mitochondrial genetic content (>5%). Additionally, uninteresting sources of variation within the data were removed. Genes removed include ribosomal structural proteins, non-coding rRNAs, HBB, and genes not expressed in ≥3 cells (Nakao et al., 2004). Datasets were subsequently analyzed using R package Seurat v2.2.0 (Satija et al., 2015). Principle Component Analysis (PCA) was employed to analyze individual samples, while Canonical Correlation Analysis (CCA) was used to analyze pooled samples (Butler et al., 2018). The number of principal components (PCs) used to cluster cells was determined by the Jackstraw method, and the number of Canonical correlation components (CCs) used was determined by manual inspection of scree plots. After identifying the number of PCs and CCs to be included for downstream analyses, a graph-based clustering approach implemented in Seurat was used to iteratively cluster cells into groups, based on similarities of those components among cells. The t-SNE method was utilized to visualize resulting clusters. The “FindAllMarkers” function in Seurat was used to identify differentially expressed genes between clusters with a fold-change of >2 and a Bonferroni adjustment of *p*-value <0.05 as a statistical significance threshold. To determine if differentially expressed genes belong to identifiable groups, pathway analysis was carried out using the Ingenuity Pathway Analysis (IPA, Qiagen Bioinformatics, Redwood City, CA, United States). Single-cell RNA sequencing data have been deposited in the NCBI Gene Expression Omnibus (GEO^1^) with the accession number GSE128243.

## Results

### Single-Cell RNA-Sequencing of Unstimulated Peripheral Blood NKT Cells Reveals Four NKT Sub-Populations

Three unstimulated NKT cell data sets were subjected to stringent quality control metrics and were subsequently combined using Seurat’s MultiCCA (Supplementary Figure 1A). A total of 5,501 NKT cells were analyzed. Based on 4,144 NKT cells that passed QC, 12,176 genes were identified across single cells, from which four cell clusters were defined using unsupervised analysis that did not rely on known markers of NKT cells, named UnstimC1 to UnstimC4 (Figures 1B–E; Supplementary Figure 2A and Supplementary Table 1). UnstimC1, the largest cluster, represented more than half of total unstimulated NKT cells (64%). Few specific gene markers were identified except for the marginal upregulation of GZMK and FOSB (Figure 1C).

**FIGURE 2:**
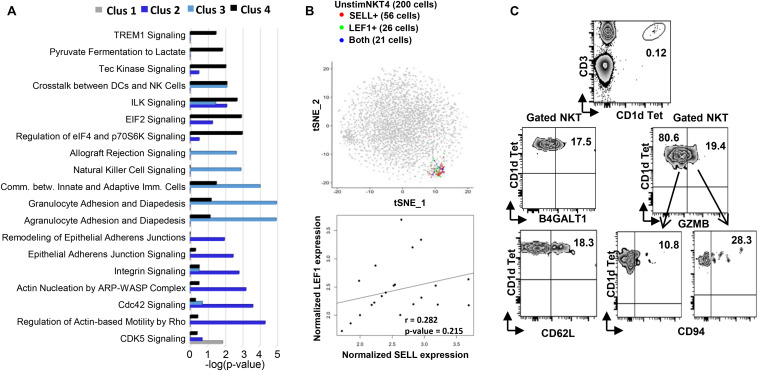
Flow cytometry and Ingenuity Pathway Analysis confirming the existence and functional prediction of the steady state human NKT clusters. **(A)** Ingenuity pathway analysis (IPA) of differentially expressed genes in individual clusters. Pathway enrichment is expressed as the –log (*p*-value) adjusted for multiple comparison **(B)** up plot: t-SNE analysis of SELL and LEF1 co-expression in unstimC4 NKT cells; lower plot: SELL and LEF1 expression correlation analysis in the cells expressing both. **(C)** Flow cytometry analysis to confirm the existence of human PBMC NKT clusters identified by scRNA-seq. Flow plots of NKT cells from PBMCs showing the expression of B4GALT1 and CD62L (SELL), respectively; and the co-expression of GZMB and CD94 (KLRD1).

Within UnstimC2 (26% of total), multiple CDC42 related genes (CDC42SE1, CDC42SE2, CDC42) were upregulated, which were reported to control T cell cytoskeleton organization, trans-endothelial migration and polarization (Rougerie and Delon, 2012). In addition, B4GALT1, a member of β-1,4-GalT family, is involved in T cell activation and intercellular contact formation (Sun et al., 2014), while PRDM1 (Blimp1) is associated with tissue resident memory T cell differentiation (Zundler et al., 2019) (Figures 1C–E). Flow cytometry analysis on PBMCs from healthy individuals showed the existence of B4GALT1^+^ NKT cells (Figure 2C). Furthermore, IPA analysis showed dramatically enriched actin-based motility signaling, integrin signaling and CDC42 signaling (Figure 2A), supporting the enhanced motility and tissue resident properties of UnstimC2 NKT cells.

Effector functions of NKT cells are not limited to cytokine production, as activated NKT cells display cytotoxic activities (Gumperz et al., 2002). UnstimC3 distinctively over expressed multiple cytotoxic-related genes, including GZMH, GZMB, FGFBP2, KLRD1, GNLY, KLRC1, and NKG7 (Figures 1C–E; Supplementary Figure 2A and Supplementary Table 1). GZMB and KLRD1 (CD94) co-expression was confirmed by flow cytometry (Figure 2C). IPA analysis verified the enrichment of natural killer cell signaling, allograft rejection signaling, granulocyte and agranulocyte adhesion and diapedesis pathways (Figure 1E). In addition, upregulated CCL4 and CCL5 may work as chemoattractant for the recruitment of natural killer (NK) cells, monocytes and T cells for a synergistic effector function and local inflammation.

Even though UnstimC4 constituted only a small proportion of total NKT cells (5%), it represented a subpopulation of NKT cells with the upregulated central memory T cell marker CD62L, LEF1 and immature maker CD79A (Figures 1C–E). Recent reports showed the stem cell-like property of CD62L^+^ central memory T cells and the proliferation and survival advantages of human CD62L^+^ NKT cells (Graef et al., 2014; Tian et al., 2016). LEF1 regulates CD127, c-myc, and Gata3 to promote NKT cell expansion and NKT2 cell effector differentiation (Carr et al., 2015), while CD79A is involved in the immature and immune suppressive phenotype maintained in naive myeloid cells (Luger et al., 2013). T-SNE analysis of SELL and LEF1 co-expression showed that majority of LEF1 expressing cells in unstimC4 co-expressed SELL, which suggest the collaborative role of LEF1 and SELL in unstimC4 NKT regulation (Figure 2B). Flow cytometry analysis on PBMCs from healthy individuals showed the expression of CD62L (SELL) in NKT cells (Figure 2C). Intriguingly, cells from this cluster showed upregulation of multiple cellular metabolism related genes, including the lactate dehydrogenase subunit B (LDHB) (Chen et al., 2016) and nitric oxide synthase interacting protein (NOSIP) (Schleicher et al., 2005; Bogdan, 2015) (Figure 1C), which may represent the metabolic machinery driving the advanced proliferation and homeostasis of these NKT cells (Tian et al., 2016). Consistent with this expectation, UnstimC4 showed enriched EIF2, p70S6K and Integrin-linked kinase (ILK) signaling (Figure 2A), which related to cell stress recovery (Shrestha et al., 2012), survival and growth (Harada et al., 2001). Overall, UnstimC4 NKT cells manifested a relative immature phenotype with unique metabolic features sustaining the advanced capacity of expansion and homeostasis.

### Single-Cell RNA-Sequencing of Stimulated Peripheral Blood NKT Cells Reveals Five NKT Sub-Populations

**FIGURE 3:**
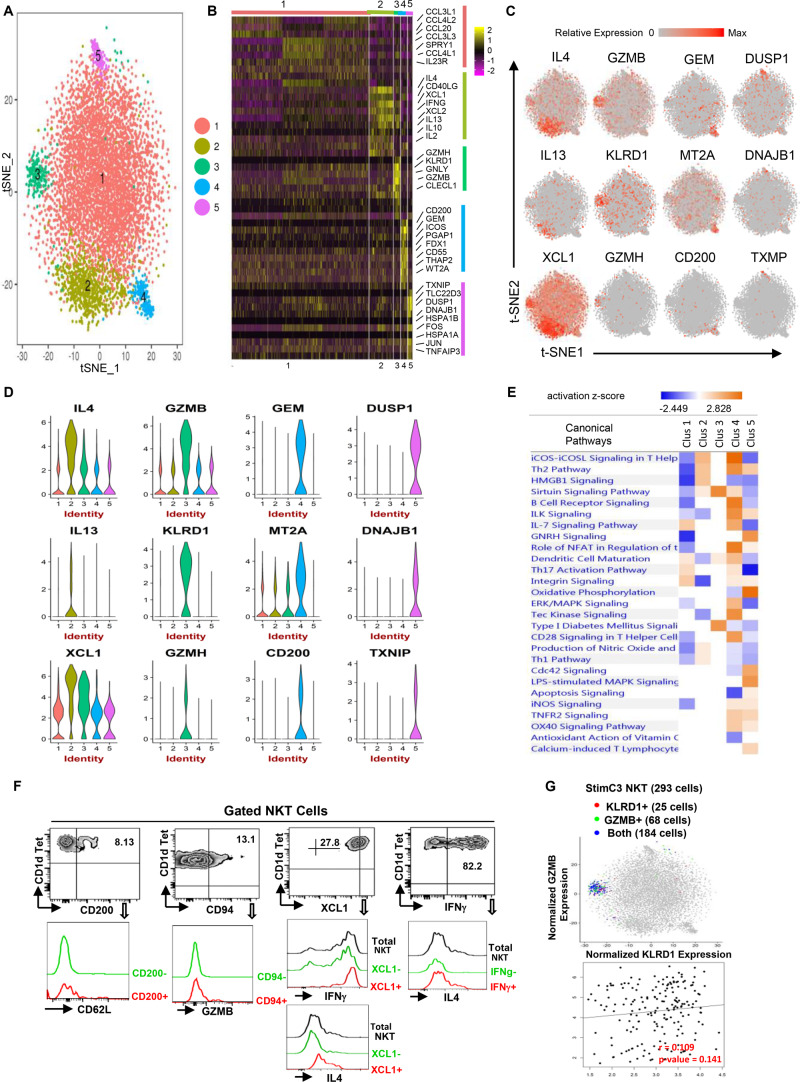
Single-cell RNA sequencing reveals the functional heterogeneity of stimulated human NKT cells. **(A)** Transcriptomic analysis on 7824 stimulated NKT cells was performed using 10X Genomics platform. t-SNE dimensionality reduction analysis identified five major clusters. **(B)** Heatmap of the top 10 most differentially expressed genes in each cluster from **(A,C,D)** Feature t-SNE plots and violin plots depicting cluster-specific single-cell gene expression of individual genes. **(E)** Ingenuity pathway analysis (IPA) of differentially expressed genes in individual clusters. Pathway enrichment in individual clusters is expressed as activation z-score shown in the heatmap. **(F)** Representative flow cytometry plots of NKT cells gated from PMA/ionomycin stimulated PBMCs, depicting the co-expression of CD200/CD62L (SELL), CD94 (KLRD1)/GZMB, XCL1/IFNG or IL4, IFNG/IL4. Data are representative of three independent experiments from different healthy individuals. **(G)** KLRD/GZMB co-expression analysis on StimC3 NKT cells, and KLRD/GZMB expression correlation analysis on NKT cells expressing both genes.

Three PMA/ionomycin stimulated NKT cell data sets were subjected to stringent quality control metrics and were subsequently combined using MultiCCA (Supplementary Figure 1B). A total of 8,618 NKT cells were analyzed. Based on 7,824 NKT cells that passed QC, 12,426 genes were identified across single cells, from which five cell clusters were defined using unsupervised analysis, named StimC1 to StimC5 (Figure 3; Supplementary Figure 2B and Supplementary Table 2).

In StimC1, the major post-stimulation NKT cluster, multiple chemokines including CCL3, CCL3L1, CCL4L2, CCL4, were upregulated, which is consistent with the result that CCL3 and CCL4 are the predominant response beside IFNγ and TNFα in stimulated human NKT cells (Snyder-Cappione et al., 2010). Interestingly, IL23R and RORC, the NKT17-signature genes, were upregulated sparsely within StimC1 (Figure 3B, Supplementary Figure 3 and Supplementary Table 2). Strong positive correlation of IL23R/RORC co-expression was identified in StimC1 NKT cells that expressed both genes (Supplementary Figure 3A). This result suggests the presence of NKT17 cells in StimC1. Nevertheless, NKT17 effector cytokines, such as IL-17 and IL-22, were not detected in any of the NKT clusters analyzed. Our result is in concordance with previous reports showing that human circulating NKT cells are intrinsically endowed with the capacity to generate IL-17, but require special proinflammatory environment to carry out this potential (Moreira-Teixeira et al., 2011; Venken et al., 2019).

StimC2 represented the NKT cells highly expressing multiple cytokines, including IL4, IFNγ, IL13, IL2, and IL10, as well as the C class chemokines XCL1 and XCL2 (Figures 3B–D and Supplementary Figure 2B). Flow cytometry analysis on PMA/Ionomycin stimulated peripheral blood NKT cells confirmed the co-expression of IFNγ/IL4, XCL1/IFNγ and XCL1/IL4 (Figure 3F).

StimC3 primarily kept the original transcriptome pattern and the proportion of UnstimC3. In addition to the cytotoxic related signature genes in UnstimC3, StimC3 showed upregulated PRF1 (perforin) and multiple chemokines including XCL1, XCL2, CCL4L1 (Figures 3B–D and Supplementary Table 2), indicating the further elevated cytotoxic and chemo attractive function post stimulation. Flow cytometry analysis of healthy individual NKT cells and t-SNE specific genes analysis showed the co-expression of KLRD1 and GZMB in StimC3 (Figures 3F,G). These data indicate that this NKT cluster maintain the original steady state cytotoxic features with enhanced chemo attractive and inflammatory regulation properties post stimulation.

In addition to SELL and CD55, StimC4 showed an extra cluster of upregulated genes compared to UnstimC4 (Figures 3B–D). CD200, the top upregulated gene in the cluster, may deliver an inhibitory signal for macrophage lineage, while MT2A, a free radical scavenger, plays a crucial pathophysiological role in anti-oxidation, anti-apoptosis and anti-inflammation (Ling et al., 2016). Flow cytometry analysis showed the co-expression of CD200 and CD62L (SELL) (Figure 3F). Interestingly, IPA analysis indicated the increased costimulatory signaling pathways including ICOS-ICOSL and CD28 signaling pathways (Figure 3E). It has been shown that ICOS is required for CD4^+^ NKT cell survival and homeostasis in the periphery in a AHR mouse model (Akbari et al., 2008), which suggests the potential involvement of ICOS-ICOSL co-stimulation signaling in the enhanced expansion and homeostasis in StimC4. Furthermore, the activated ILK and Tec kinase, but inhibited apoptosis and oxidative phosphorylation signaling pathways (Figure 3E), supported the overall enhanced cell growth, proliferation and survival capacities of StimC4 NKT cells (Liu et al., 2005; Chang et al., 2013).

StimC5, a unique post-stimulation NKT cluster, showed the upregulation of multiple immediate early genes (IEGs) (Figures 3B–D). including FOS, JUN, FOSB, DNAJB1, DUSP1, EGR1, and HSPs. IEGs are involved in multiple gene regulation program and T cell short term biochemical memory formation (Clark et al., 2011). Other than IEGs, the top two in the upregulated gene list, Thioredoxin-interacting protein (TXNIP) and Glucocorticoid-induced leucine zipper (TLC22D3), repress cellular glucose uptake and regulate T-cell receptor activation, respectively (Muri et al., 2018). IPA analysis showed upregulated apoptosis, oxidative phosphorylation and iNOS signalings, whereas down regulated CD28 and iCOS-iCOSL costimulatory signaling pathways in StimC5 NKT cells. iNOS signaling controls the susceptibility of activated human T cells to death by neglect to determine the level of persisting T cell memory (Vig et al., 2004). Absence of efficient costimulatory signals results in T cell anergy, while exhausted T cells show changed metabolism pattern and favor oxidative metabolism (Wherry et al., 2007; MacIver et al., 2013). Therefore, StimC5 may represent an NKT population at the early or transitional stage of exhaustion or anergy post stimulation.

### Sub-Cluster Analysis on Cytokine Producing NKT Cells

**FIGURE 4:**
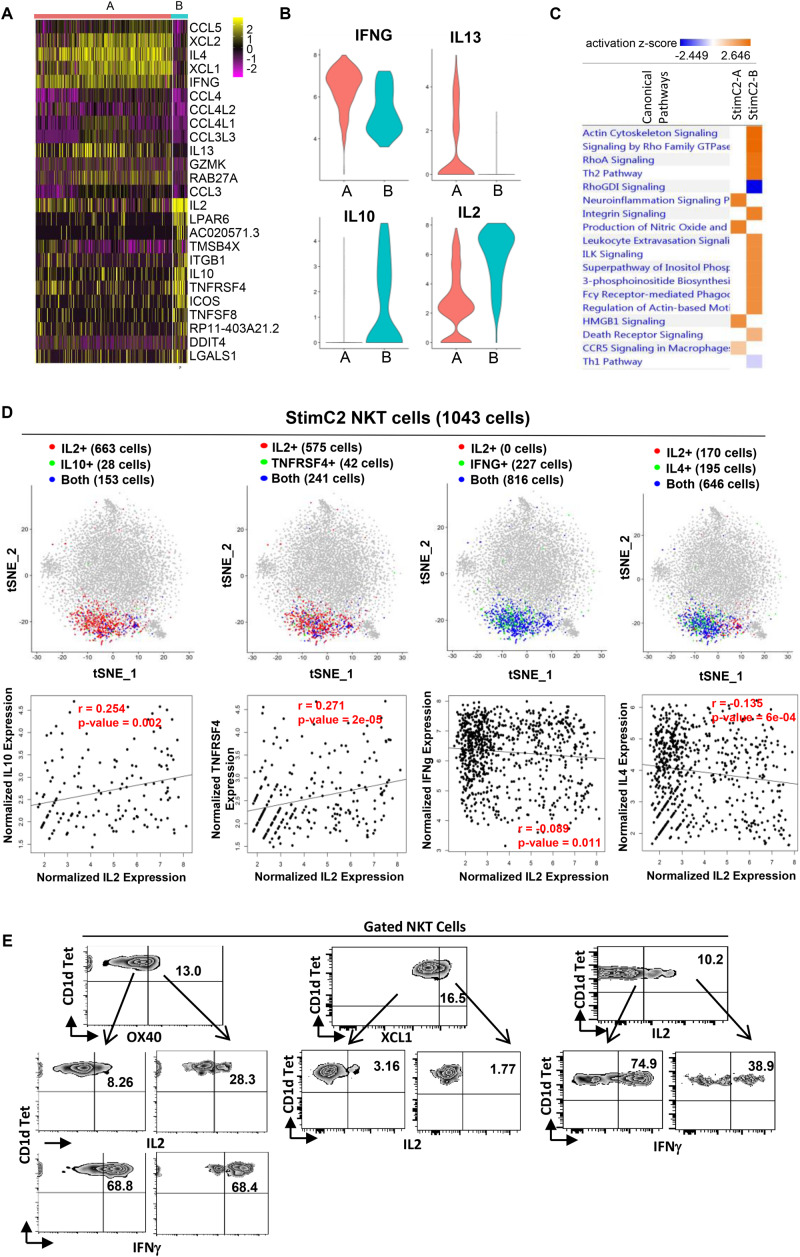
Sub-cluster of stimulated cytokines expressing NKT cells showed regulatory T cell phenotype. **(A)** Heatmap of the discriminative gene sets defining the two sub-clusters within the stimulated cluster two NKT cells. **(B)** Violin plots showing the expression distribution of candidate genes across two sub-clusters of NKT cells (orange for StimC2-A; blue for StimC2-B). **(C)** Ingenuine pathway analysis (IPA) of differentially expressed genes in the two sub-cluster NKT cells. Pathway enrichment in individual clusters is expressed as activation *z*-score shown in the heatmap. **(D)** The IL2/IL10, IL2/TNFRSF4, IL2/IFNγ and IL2/IL4 gene pair co-expression analysis (upper panel), and the gene expression correlation analysis (lower panel) in cells expressing both genes in StimC2 NKT cells. **(E)** Representative flow cytometry plots of NKT cells gated from PMA/ionomycin stimulated PBMCs, depicting the co-expression of OX40 (TNFRSF4)/IL2; OX40 (TNFRSF4)/IFNγ; XCL1/IL2; IL2/IFNγ.

Based on the gene expression patterns, StimC2 can be further separated into two sub-clusters, in which sub-cluster1 (StimC2-A) was represented by NKT cells highly expressing XCL1, XCL2, IFNγ, IL4, and IL13, whereas sub-cluster2 (StimC2-B) exhibits high expression of IL2, IL10, TNFRSF4 (OX40) and ICOS (Figures 4A,B and Supplementary Table 3). To analyze these potential NKT functional lineages, we evaluated the co-expression and expression correlation of different cytokine and related gene pairs. Even though IL2/IL10, IL2/TNFRSF4 (OX40), IL2/IFNγ and IL2/IL4 are all co-expressed in large numbers of NKT cells within StimC2, IL2/IL10, IL2/TNFRSF4 expression were positively correlated, whereas IL2/IFNγ and IL2/IL4 were instead negatively correlated (Figure 4D). Consistent with this expression correlation pattern, flow cytometry analysis on stimulated NKT cells showed favorable co-expression in OX40/IL2, but not in OX40/IFNγ, IL2/XCL1 and IL2/IFNγ (Figure 3E), which further confirmed the positive correlation of OX40/IL2, but negative correlation of IL2/IFNγ at the protein level. These data indicated the presence of two sub-clusters (StimC2-A and StimC2-B) inside the post stimulation cytokine producing NKT cells. The StimC2-B (IL2^+^IL10^+^OX40^+^ICOS^+^IL4^–^IFNγ^–^XCL^–^) may represent the NKTreg subset with the transcriptome featuring the IL10 producing regulatory NKT cells identified in mouse (Sag et al., 2014). Meanwhile, multiple upregulated genes in StimC2-B cluster are among the gene list specifically expressed in human PBMC Treg and/or mouse spleen Tregs, such as TNFRSF4 (OX40), ICOS, S100A4, ITGB1, IL2RA (Zemmour et al., 2018). Furthermore, StimC2-B showed highly activated integrin signaling, actin cytoskeleton signaling, and Rho family signaling (Figure 4C). Rho signaling is critical for Treg induction and suppression function (Kalim et al., 2016), while actin remodeling is required for not only sustained TCR signaling (Miletic et al., 2003), but also immune synapse stability and mobility, which is critical for Treg induction (Diana et al., 2011). Therefore, these results support the idea that StimC2-B may represent a special group of human NKT cells with regulatory function.

### Integrative Analyses of Unstimulated Versus Stimulated Human NKT Cells

In order to clarify the relationship of different clusters of unstimulated and stimulated NKT cells, an integrated data analysis on merged unstimulated and stimulated NKT cell data was performed. A total of seven NKT clusters were identified from merged data, in which cluster five was composed of UnstimC3 and StimC3; while Cluster 6 were composed of UnstimC4 and StimC4 (Supplementary Figure 4). This result indicates that the overall gene expression pattern of UnstimC3 (cytotoxic) and UnstimC4 (immature) clusters of NKT cells remain relative stable post stimulation (StimC3 and StimC4), respectively. It is not surprising that StimC1 and StimC2 deviated from the unstimulated major clusters due to the respective dramatically upregulated chemokine or cytokine expressions. Interestingly, UnstimC2-specific gene cluster remain expressed in stimulated NKT cells without an obvious trend of preferential to either StimC1 or StimC2 (data not shown), indicating that NKT cells with tissue resident potential have no clear biased trend of either chemokine or cytokine production.

### Comparison of the scRNA-Seq Based Classification With Traditional Classifications of Human NKT Cells

In the current study, we explored and assessed the peripheral blood human NKT cell diversity in an unbiased and more comprehensive way based on the scRNA-seq data. Human NKT cells have traditionally be classified based on cell surface costimulatory receptors such as CD4 and CD8, which is linked to NKT cell functions (Gumperz et al., 2002; Lee et al., 2002). To make a comparison or connections between the scRNA-seq-based and traditional NKT classifications, we first evaluated the CD4 and/or CD8 transcriptome levels in different clusters from both steady state and stimulated NKT cells. Nevertheless, none CD4 nor CD8 were identified to be detectable in any cluster of NKT cells, which could result from the low and limited capture efficiency and high dropout, a common short come of the current scRNA-seq platform (Haque et al., 2017). To circumvent the situation, we performed the flow cytometry analysis of CD4 and related cluster-specific signature gene expressions on the PBMCs from healthy individuals. As shown in Supplementary Figure 5A, 45% of NKT cells in steady state expressed CD4, which is within the range (15–80%) of CD4^+^ in human peripheral NKT cells (Krijgsman et al., 2018). Within the CD62L (SELL)^+^ NKT cells, the representative of UnstimC3, 64% of them expressed CD4; whereas, only 7.69% of CD94 (KLRD)^+^, the representative of UnstimC4 NKT cells, expressed CD4 (Supplementary Figure 5A). This result is consistent with a previous study showing that CD94 were nearly exclusively expressed by CD4^–^ human NKT cells (Lee et al., 2002). Post PMA/ionomycin stimulation, CD94^+^, the representative of StimC3, and the CD62L^+^, the representative of StimC4, NKT cells kept the similar trend of CD4 expression pattern (Supplementary Figure 5B). In addition, within the CD200^+^ NKT cells, another representative for StimC3, majority of them (71.7%) expressed CD4. Overall, our result indicated that UnstimC3 and StimC3 are composed of more CD4^+^ than CD4^–^ NKT cells. In consistent with previous reports, both CD4^+^ and CD4^–^ NKT cells produced IL4 and IFNγ, whereas relative higher frequencies of CD4^+^ NKT cells were in IL4 producing NKT cells than that in IFNγ producing NKT cells (Gumperz et al., 2002; Lee et al., 2002). As the signature genes of the StimC2-B NKT cells in StimC2, IL2 and OX40 (TNFRSF4) were confirmed to be expressed in stimulated NKT cells by flow cytometry. Within the IL2^+^ or OX40^+^ NKT cells, about 60-70% of them expressed CD4, indicating that this group of NKT cells are composed of more CD4^+^ than CD4^–^ NKT cells (Supplementary Figure 5B).

## Discussion

Murine RORγt^+^ IL-17 producing NKT (NKT17) cells from thymus and peripheral immune organs have been extensively studied but the prevalence and phenotype of their human counterparts is poorly understood. RORγt and interleukin-23 receptor (IL-23R) are ubiquitously expressed in both adaptive and innate IL17-producing immune cells including NKT cells (Gaffen et al., 2014). IL-23 is necessary for the terminal differentiation and inflammatory functions associated with T helper-17 cells (Th17). In mice, NKT17 cells are very scarce in peripheral circulation and the majority of NKT17 cells seem to be tissue resident cells that can be found in lung tissue and lymph nodes (Lynch et al., 2015). Mouse NKT 17 cells do not or only modestly recirculate but instead show long-term residence at peripheral tissues. Even though our unbiased scRNA-seq data analysis on human peripheral blood did not show a unique cluster of RORC expressing NKT cells, we did observe the RORC and/or IL23R expressing NKT cells distributed inside StimC1. The co-expression and strong positive correlation of RORC and IL23R in these cells highlighted the existence of NKT17 cells in peripheral blood even though no IL17 expression was observed. Given the requirement of proinflammatory environment for human NKT17 cell effector phenotype and function (Moreira-Teixeira et al., 2011), our data suggest that these NKT cells might represent a transitional phenotype of NKT17 cells, prior to migration into peripheral tissues, where they will further differentiate under influence of the local microenvironment and signaling cues.

Multiple reports have showed the direct cytotoxic effect of activated human NKT cells, such as direct antitumor cytotoxicity (Metelitsa et al., 2001), cytotoxicity toward Treg in allergic asthma development (Nguyen et al., 2008) and the cytolysis of the *Leishmania infantum* infected immature DCs (Campos-Martin et al., 2006). However, it remains unclear whether cytotoxicity is a common effector function of all activated NKT cells, or it belongs to a specific NKT population endowed with this specific effector function, and the related molecular mechanisms of the cytotoxic property. Our data clearly showed that a small group of peripheral blood NKT cells highly express genes related to cytotoxic function even at steady state and keeps the identity post activation, highlighting at least the existence of a subset of NKT cells that inherit the privilege of professional killer cells with direct and indirect cytotoxic properties. In addition to the canonical perforin/granzyme mediated cytotoxic effector function manifested by UnstimC3 and StimC3 NKT cells, our result does not eliminate other possible cytotoxic mechanisms performed by NKT cells, such as FAS/FASL dependent cytotoxic function (Wingender et al., 2010). The role of this cluster of NKT cells in different peripheral tissues and disease conditions remains to be explored in the future.

The strong influence on immune response of NKT cells of such a small population and a nearly monospecific TCR repertoire come from the contextual regulation of the multiple subsets and effector functions of NKT cells. In both humans and mice, NKT cells can be separated into CD4^+^ and CD4^–^ populations. The expression of CD4 on human NKT cells has been used as a useful predictor of CD4^+^ NKT cells with the potential to generate more Th2-type cytokines with relative suppressive phenotype, in contrast to proinflammatory CD4^–^ NKT cells (Gumperz et al., 2002; Lee et al., 2002). Through evaluating the co-expression of CD4 with cluster specific signature genes by flow cytometry, we concluded that the cytotoxic NKT cluster (UnstimC3 and StimC3) are almost exclusively CD4^–^, whereas the immature NKT cluster (UnstimC4 and StimC4), and regulatory StimC2-B showed relatively higher CD4 expression compared to total NKT population. These results support the overall anti-inflammatory versus pro-inflammatory identities on human peripheral blood NKT cell classified based on CD4 expression. Nevertheless, our study supplies a more delicate and comprehensive human NKT classifications which is transcriptome based, unbiased and function related. These cluster-specific signature genes supply extra markers other than CD4 and CD8 for more comprehensive and accurate human NKT cell classification.

Overall, using single-cell RNA sequencing and unbiased genomic classification followed by flow cytometry profiling, our study provides a general model for human peripheral blood NKT cell identity and heterogeneity. Our study reveals the presence of multiple specific NKT cell clusters including a cluster with specific cytotoxic capacity, a cluster with advanced proliferation and survival but immature phenotype, as well as an NKT sub-cluster with potential regulatory properties in steady state and stimulated peripheral blood NKT cells (Supplementary Table 4). Further functional confirmation and molecular mechanism exploration of the homeostasis and functional activities of these NKT subsets will eventually lead the way to tailored therapies that target selected NKT subsets.

## Data Availability Statement

The datasets generated for this study can be found in the NCBI Gene Expression Omnibus with the accession number GSE128243.

## Ethics Statement

The studies involving human participants were reviewed and approved by The Institutional Review Board at Henry Ford Health System. The patients/participants provided their written informed consent to participate in this study.

## Author Contributions

LZ and Q-SM conceived and designed the study. IA analyzed single-cell RNA sequencing data. JW performed NKT sorting and flow cytometry analysis. XW prepared single-cell cDNA library. ID performed single cell sequence processing with 10× Cell Ranger and Ingenuity Pathway Analysis; LZ, IA, and Q-SM wrote the manuscript, which was commented on by all authors.

## Conflict of Interest

The authors declare that the research was conducted in the absence of any commercial or financial relationships that could be construed as a potential conflict of interest.

## Footnotes

1 http://www.ncbi.nlm.nih.gov/geo/

## Abbreviations

NKT: Natural killer T
PBMCs: Peripheral blood mononuclear cells
scRNA-seq: single-cell RNA sequencing.
