# Supplementary material for: Single-Cell RNA-Seq Analysis Uncovers Distinct Functional Human NKT Cell Sub-Populations in Peripheral Blood
Source: Front Cell Dev Biol. 2020 May 26;8:384. doi: 10.3389/fcell.2020.00384 (PMC7264113; doi:10.3389/fcell.2020.00384)
Supplement: Supplementary file 1 [file Data_Sheet_1.PDF]

Supplementary Materials for

Single-cell RNA-seq reveals distinct functional human peripheral blood NKT cell sub-populations

Li Zhou*, Indra Adrianto, Jie Wang, Xiaojun Wu, Indrani Datta, Qing-Sheng Mi*

*Correspondence to: L.Z. (lzhou1@hfhs.org) or Q.S.M. (qmi1@hfhs.org)

This PDF file includes:

Supplementary Figure 1. The canonical correlation analysis (CCA) to align three data sets of unstimulated- and stimulated-NKT cells.

Supplementary Figure 2. Other Cluster-specific genes in unstimulated and stimulated human NKT cells.

Supplementary Figure 3. RORC and IL23R co-expression and expression correlation analysis on stimC1 NKT cells. Supplementary

Supplementary Figure 4. Integrative analyses of stimulated vs. unstimulated human NKT cells.

Supplementary Figure 5. Flow cytometry analysis of cluster-specific signature molecules and CD4 co-expression in unstimulated and stimulated NKT cells.

Supplementary Table 1. The top 30 cluster discriminatory genes in unstimulated human NKT cells.

Supplementary Table 2. The top 30 cluster discriminatory genes in stimulated human NKT cells.

Supplementary Table 3. The subset discriminatory genes in Cluster2 of stimulated human NKT cells.

Supplementary Table 4. Summary of features of human NKT cell clusters

Supplementary Figure 1

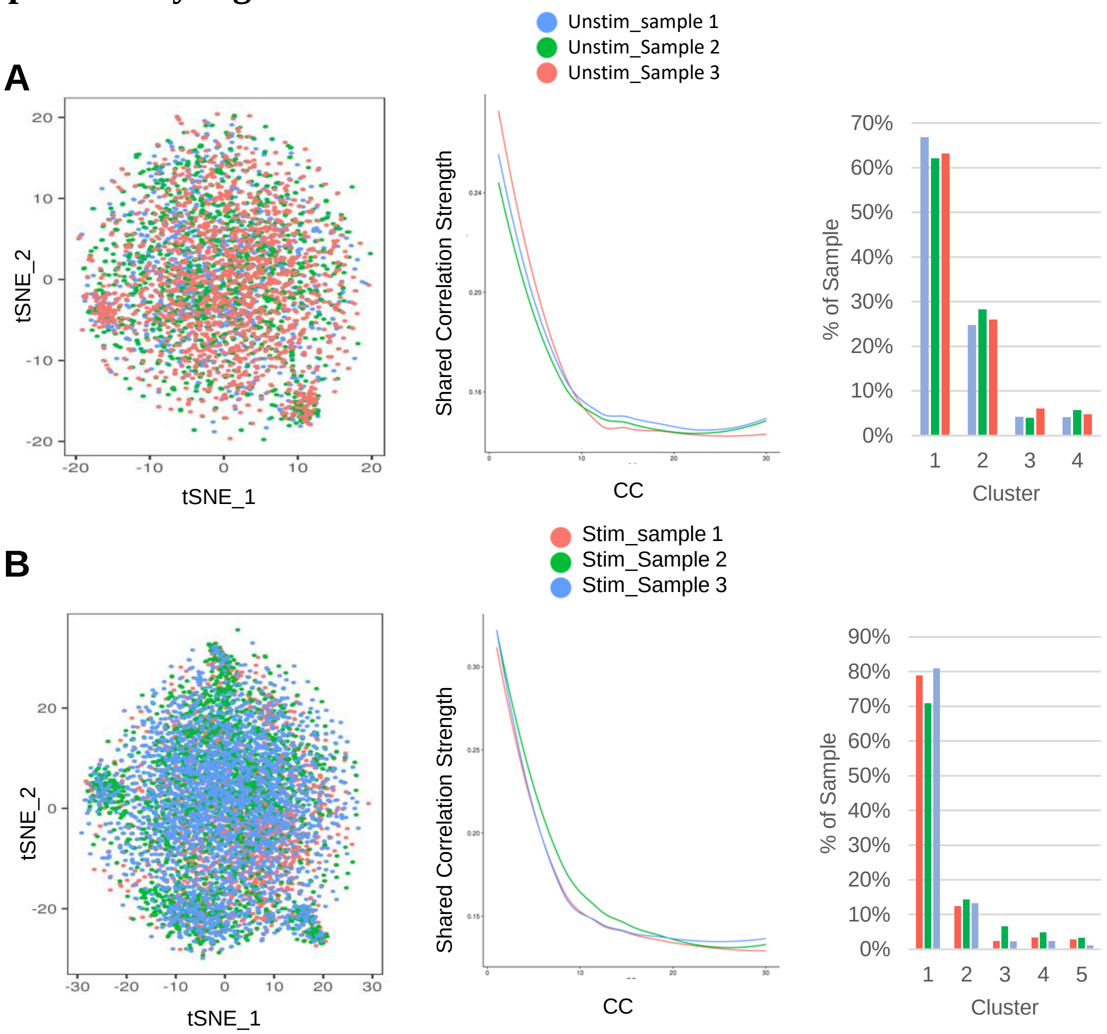

Supplementary Figure 1. The canonical correlation analysis (CCA) to align three data sets of unstimulated- and stimulated-NKT cells. The significance and variance of each data sets from unstimulated- (A) and stimulated-NKT cells (B) were evaluated and visualized using T-distributed stochastic neighbor embedding (t-SNE) plots (left panel), plotting eigenvalues in a scree plot (middle panel), and the percentage of each sample represented in each cluster indicating the clusters were evenly composed from the three individual samples (right panel).

Supplementary Figure 2

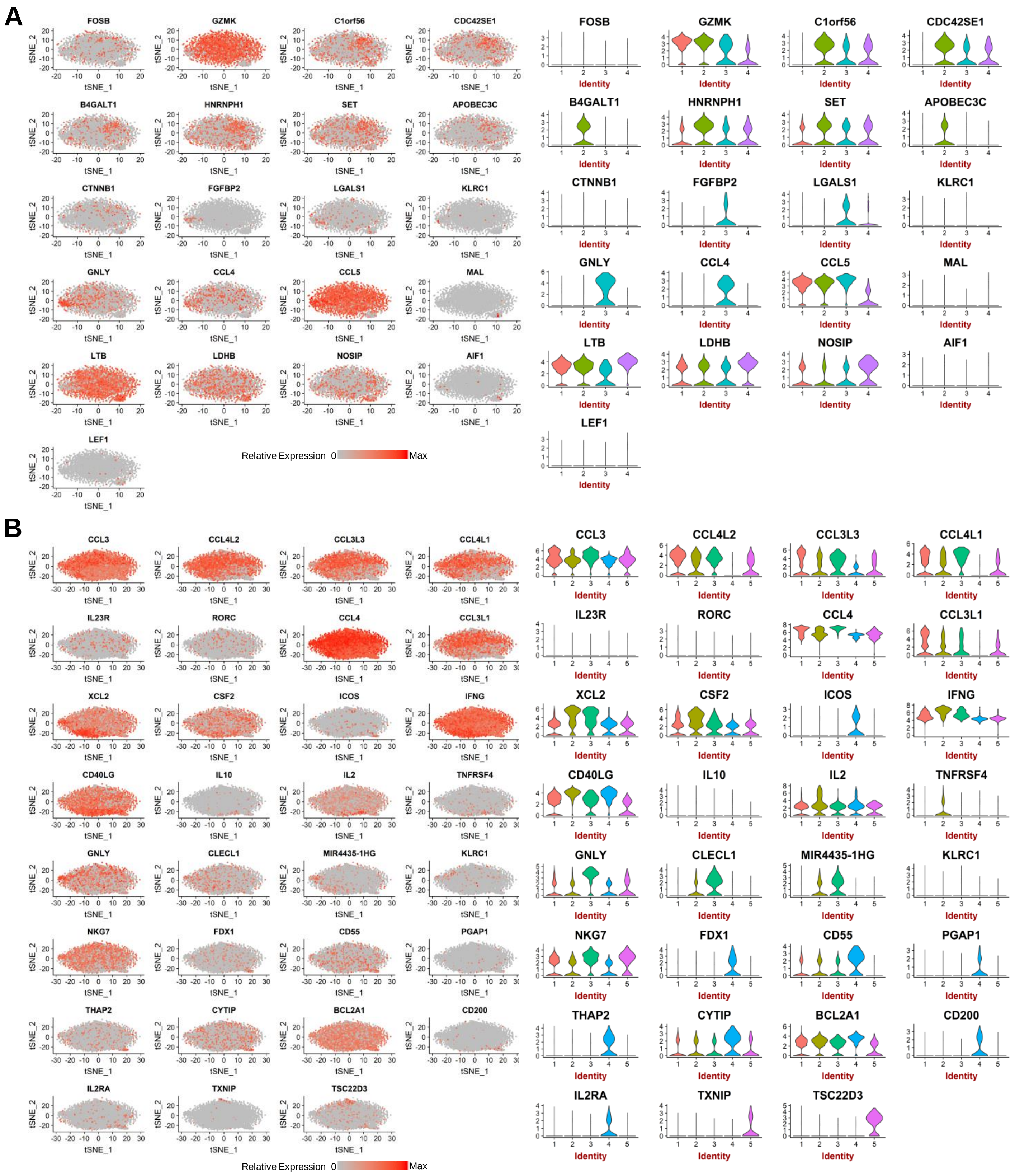

Supplementary Figure 2. Other Cluster-specific genes in unstimulated and stimulated human NKT cells. Feature t-SNE plots (left panels) and violin plots (right panels) of cluster-defining genes in unstimulated (A) and stimulated (B) human NKT cells.

Supplementary Figure 3

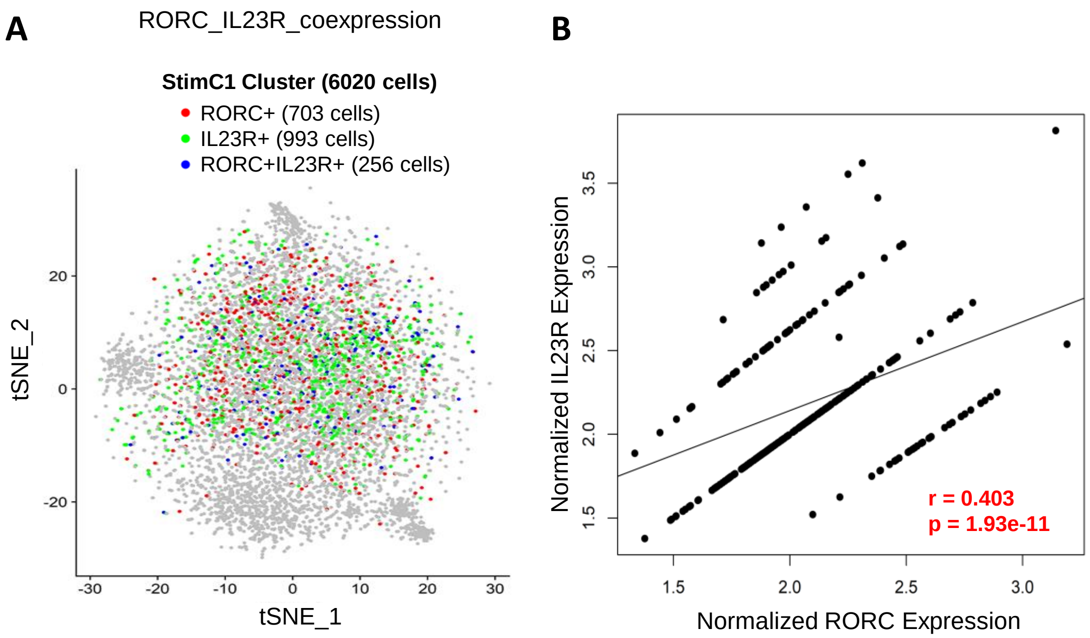

Supplementary Figure 3. RORC and IL23R co-expression and expression correlation analysis on stimC1 NKT cells. (A) t-SNE RORC/IL23R co-expression analysis on stimC1 NKT cells. (B) RORC/IL23R expression correlation analysis on stimC1 NKT cells expression both genes.

Supplementary Figure 4

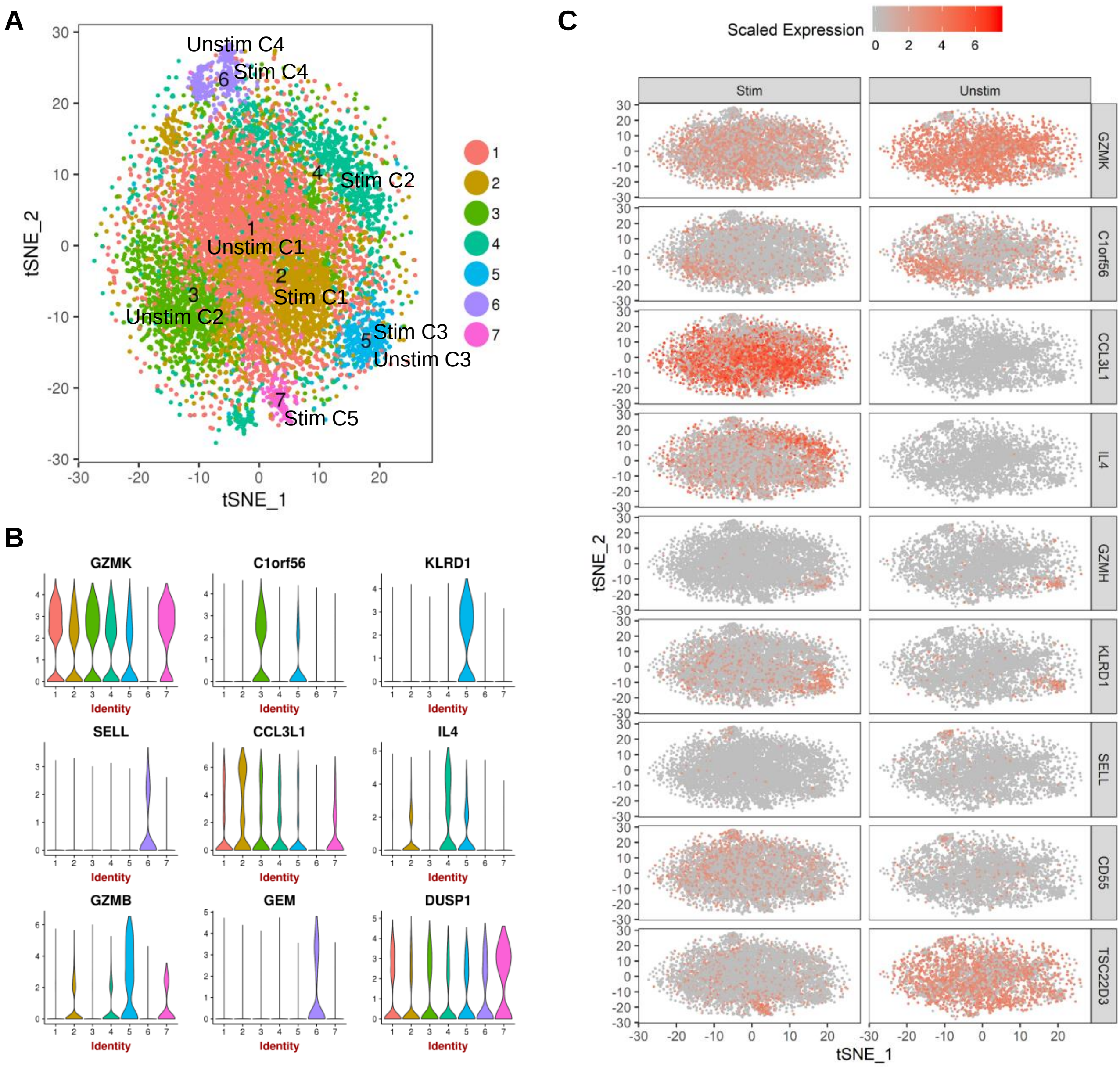

Supplementary Figure 4. Integrative analyses of stimulated vs. unstimulated human NKT cells. (A) Transcriptomic analysis on combined unstimulated and stimulated NKT cells was performed using 10X Genomics platform. t-SNE dimensionality reduction analysis identified 7 major clusters. (B) Violin plots depicting cluster-specific genes from unstimulated (GZMK, C1orf56, KLRD1, and SELL for unstimulated clusters 1, 2, 3, and 4, respectively) and stimulated (CCL3L1, IL4, GZMB, GEM and DUSP1 for stimulated clusters 1, 2, 3, 4 and 5, respectively) human NKT cells. (C) Feature plots comparing cluster-specific genes in stimulated and unstimulated NKT cells.

Supplementary Figure 5

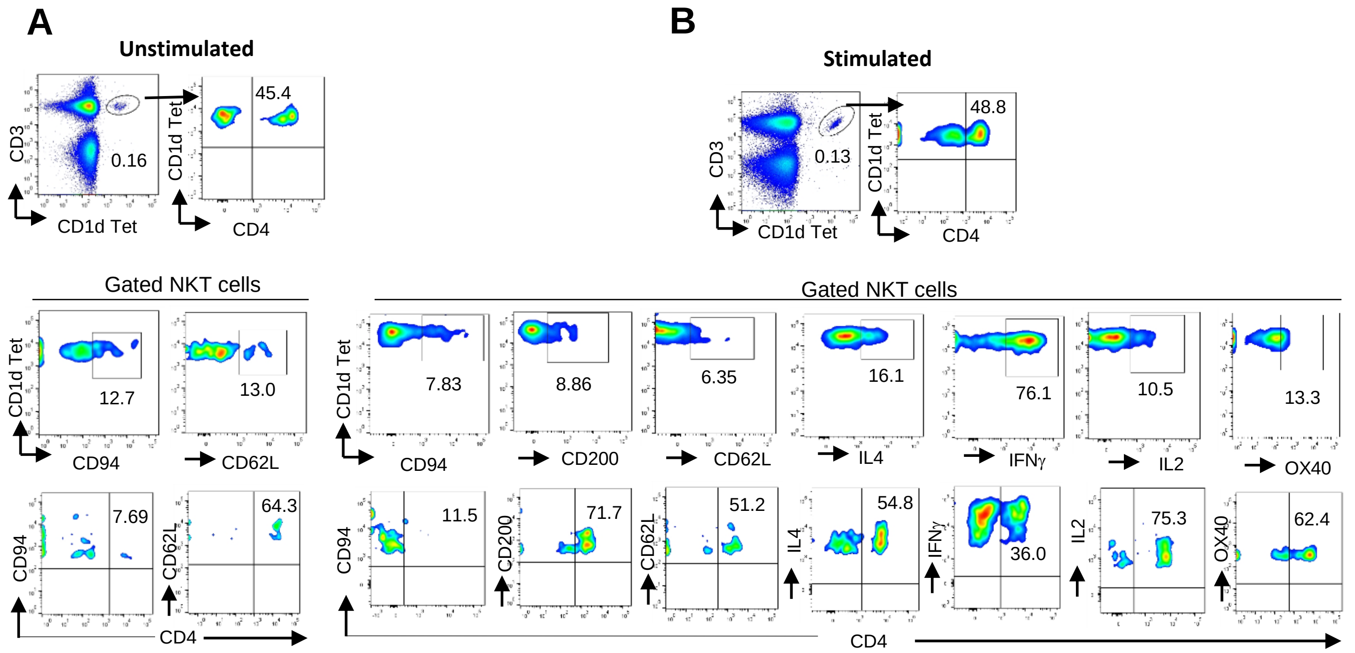

Supplementary Figure 5. Flow cytometry analysis of cluster-specific signature molecules and CD4 co-expression in unstimulated and stimulated NKT cells. (A) co-expression analysis of CD4 and unstimC3 (CD94), unstimC4 (CD62L) signature molecules in unstimulated human peripheral blood NKT cells. (B) co-expression analysis of CD4 and StimC3 (CD94), StimC4 (CD200, CD62L), StimC2 (IL4, IFNγ), StimC2_subclus2 (IL2, OX40) signature molecules in human peripheral blood NKT cells stimulated with PMA/ionomycin.

Supplementary Table 1. The top 30 cluster discriminative genes in unstimulated human NKT cells

Gene	log (Fold-Change)	% Cells inside Cluster	% Cells outside Cluster	p_value	Adjusted p-value	Cluster
GZMK	0.267	0.922	0.75	4.12E-33	5.02E-29	1
FOSB	0.275	0.177	0.111	1.26E-08	0.00015391	1
C1orf56	1.259	0.708	0.231	4.09E-198	4.99E-194	2
CDC42SE1	1.217	0.676	0.233	2.18E-178	2.65E-174	2
B4GALT1	1.179	0.556	0.137	3.91E-170	4.77E-166	2
HNRNPH1	1.051	0.714	0.307	5.19E-154	6.32E-150	2
APOBEC3C	0.941	0.371	0.113	2.69E-85	3.28E-81	2
C16orf54	0.791	0.433	0.177	3.99E-69	4.86E-65	2
SET	0.683	0.576	0.319	1.41E-62	1.72E-58	2
TNRC6B	0.771	0.418	0.198	9.43E-53	1.15E-48	2
MDM4	0.786	0.267	0.086	1.63E-52	1.98E-48	2
PRDM1	0.691	0.264	0.108	3.34E-36	4.07E-32	2
CTNNB1	0.590	0.197	0.074	8.04E-30	9.80E-26	2
TMEM120B	0.615	0.177	0.064	3.92E-29	4.77E-25	2
CDC42	0.365	0.695	0.56	1.38E-28	1.68E-24	2
EIF5A	0.571	0.41	0.255	5.09E-28	6.20E-24	2
GIGYF1	0.574	0.138	0.042	6.17E-28	7.51E-24	2
CDC42SE2	0.449	0.513	0.367	9.00E-24	1.10E-19	2
RASSF3	0.534	0.148	0.066	2.98E-17	3.63E-13	2
ZNF652	0.455	0.116	0.045	7.68E-17	9.36E-13	2
TRA2A	0.479	0.24	0.138	3.38E-16	4.12E-12	2
TSPYL1	0.409	0.323	0.21	1.52E-15	1.85E-11	2
CAPZA1	0.369	0.362	0.271	1.07E-11	1.31E-07	2
RQCD1	0.357	0.128	0.064	1.68E-11	2.05E-07	2
PPP3CA	0.362	0.203	0.122	1.84E-11	2.24E-07	2
PPP1CB	0.339	0.356	0.257	1.94E-11	2.37E-07	2
SERPINB9	0.336	0.116	0.057	1.38E-10	1.69E-06	2
CBX6	0.329	0.199	0.124	2.96E-10	3.61E-06	2
RHOF	0.360	0.253	0.18	1.22E-08	0.00014831	2
TMED4	0.327	0.206	0.136	1.58E-08	0.00019236	2
SAR1A	0.344	0.253	0.18	2.87E-08	0.00035003	2
RBM6	0.340	0.117	0.069	4.14E-07	0.00504454	2
GZMH	2.209	0.517	0.013	3.14E-295	3.82E-291	3
FGFBP2	1.685	0.259	0.004	2.50E-175	3.05E-171	3
KLRD1	1.899	0.448	0.028	9.97E-166	1.21E-161	3

GZMB	1.650	0.224	0.006	3.19E-127	3.88E-123	3
GNLY	2.166	0.632	0.173	6.61E-73	8.06E-69	3
ZNF683	0.813	0.109	0.003	7.30E-62	8.90E-58	3
NKG7	0.620	0.98	0.87	1.22E-31	1.49E-27	3
CCL4	0.880	0.557	0.22	1.30E-30	1.58E-26	3
LGALS1	1.017	0.343	0.1	5.47E-28	6.66E-24	3
KLRC1	0.872	0.109	0.012	1.25E-26	1.52E-22	3
CCL5	0.446	0.97	0.887	2.81E-20	3.42E-16	3
CD52	0.377	1	0.949	1.18E-17	1.44E-13	3
AKR1C3	0.663	0.119	0.023	3.06E-16	3.72E-12	3
HLA-DRB1	0.640	0.134	0.03	2.39E-15	2.91E-11	3
LINC00152	0.678	0.318	0.141	5.60E-13	6.82E-09	3
CD300A	0.478	0.139	0.043	6.57E-10	8.00E-06	3
C12orf75	0.494	0.512	0.325	9.16E-10	1.12E-05	3
ITGB2	0.410	0.542	0.387	7.85E-08	0.00095589	3
MIR4435-1HG	0.418	0.119	0.04	1.35E-07	0.00164598	3
HOPX	0.384	0.517	0.358	6.85E-07	0.00834609	3
TES	0.483	0.154	0.065	8.85E-07	0.01078535	3
SPON2	0.478	0.119	0.045	9.97E-07	0.01214608	3
C14orf119	0.433	0.119	0.049	6.82E-06	0.08301891	3
TADA3	0.408	0.114	0.045	7.33E-06	0.08928246	3
GLIPR2	0.381	0.249	0.139	2.57E-05	0.31319003	3
CTSD	0.415	0.189	0.1	3.82E-05	0.46484328	3
HLA-DPB1	0.456	0.219	0.128	0.000107636	1	3
ABI3	0.386	0.154	0.079	0.000169125	1	3
DBI	0.401	0.408	0.305	0.000332795	1	3
S100B	0.605	0.184	0.109	0.000537984	1	3
CD79A	1.760	0.285	0.004	1.54E-194	1.88E-190	4
MAL	1.029	0.185	0.002	5.02E-131	6.12E-127	4
SELL	1.536	0.385	0.028	1.11E-124	1.35E-120	4
LEF1	1.119	0.235	0.013	3.46E-89	4.21E-85	4
FHIT	0.753	0.14	0.01	2.82E-44	3.44E-40	4
NGFRAP1	0.651	0.11	0.006	9.89E-43	1.21E-38	4
AIF1	0.816	0.135	0.011	4.10E-41	4.99E-37	4
TCEA3	0.643	0.13	0.01	1.36E-39	1.65E-35	4
LTB	0.776	0.975	0.858	5.69E-39	6.93E-35	4
CCR7	0.748	0.14	0.013	1.54E-36	1.88E-32	4
LDHB	0.811	0.815	0.508	3.67E-33	4.47E-29	4
NELL2	0.650	0.125	0.014	7.91E-28	9.64E-24	4
NOSIP	0.785	0.645	0.344	4.24E-24	5.16E-20	4
C1orf162	0.730	0.215	0.048	2.27E-23	2.77E-19	4

CD55	0.991	0.245	0.072	7.34E-20	8.93E-16	4
CAPG	0.597	0.11	0.016	1.45E-19	1.77E-15	4
USP10	0.741	0.125	0.021	3.61E-19	4.40E-15	4
IFITM1	0.579	0.855	0.658	4.08E-18	4.98E-14	4
TRAT1	0.650	0.55	0.29	3.42E-16	4.17E-12	4
ARID5B	0.763	0.185	0.051	1.47E-15	1.79E-11	4
CORO1B	0.672	0.335	0.136	1.61E-14	1.96E-10	4
TMEM156	0.523	0.21	0.069	6.25E-13	7.62E-09	4
ADAM19	0.563	0.115	0.027	4.01E-12	4.88E-08	4
LGALS1	0.765	0.25	0.105	9.81E-11	1.20E-06	4
PIM2	0.530	0.225	0.088	1.59E-10	1.93E-06	4
ITGB1	0.727	0.24	0.104	5.19E-10	6.32E-06	4
LIMS1	0.554	0.2	0.078	8.90E-10	1.08E-05	4
TMEM123	0.577	0.445	0.257	1.84E-09	2.25E-05	4
ITM2A	0.537	0.45	0.276	1.92E-08	0.00023441	4
CRIP1	0.560	0.645	0.52	6.25E-07	0.00761279	4

Supplementary Table 2. The top 30 cluster discriminative genes in stimulated human NKT cells

Gene	Log (Fold-Change)	% Cells inside Cluster	% Cells outside Cluster	p-value	Adjusted p-value	Cluster
KLRB1	0.477	0.959	0.814	2.73E-159	3.39E-155	1
CCL3L1	1.225	0.681	0.421	4.05E-118	5.04E-114	1
CCL4L2	0.705	0.821	0.604	2.52E-114	3.13E-110	1
CCL20	1.349	0.803	0.703	4.23E-81	5.25E-77	1
CCL3L3	1.189	0.625	0.494	3.75E-68	4.65E-64	1
SPRY1	1.061	0.286	0.092	7.66E-68	9.52E-64	1
CCL4	0.414	1	0.998	3.03E-64	3.77E-60	1
CXCR4	0.422	0.75	0.598	8.61E-61	1.07E-56	1
CCL4L1	0.506	0.697	0.523	2.03E-53	2.52E-49	1
GPR183	0.437	0.842	0.729	7.61E-52	9.46E-48	1
IL23R	0.697	0.207	0.058	1.62E-49	2.01E-45	1
CLEC2B	0.473	0.598	0.427	4.70E-48	5.84E-44	1
GPR171	0.438	0.65	0.504	4.33E-45	5.38E-41	1
CRTAM	0.585	0.346	0.188	4.50E-41	5.59E-37	1
AQP3	0.713	0.231	0.093	1.77E-39	2.20E-35	1
PRF1	0.453	0.441	0.275	2.00E-39	2.49E-35	1
APOBEC3G	0.391	0.503	0.353	1.33E-34	1.65E-30	1
IFNGR1	0.487	0.254	0.134	5.49E-29	6.83E-25	1
GPR65	0.426	0.372	0.244	1.61E-27	2.00E-23	1
CCL3	0.655	0.948	0.952	9.93E-27	1.23E-22	1
NCR3	0.386	0.291	0.166	3.34E-26	4.15E-22	1
IL4I1	0.469	0.148	0.055	2.76E-25	3.43E-21	1
RORC	0.423	0.159	0.067	6.44E-24	8.01E-20	1
ATM	0.373	0.241	0.134	6.23E-23	7.74E-19	1
GTF3C1	0.433	0.135	0.052	1.99E-22	2.47E-18	1
SKIL	0.351	0.447	0.344	2.75E-21	3.41E-17	1
GYG1	0.396	0.162	0.075	4.67E-21	5.80E-17	1
KLRG1	0.352	0.244	0.142	6.06E-21	7.52E-17	1
PLAUR	0.470	0.238	0.153	1.59E-16	1.98E-12	1
B4GALT1	0.370	0.239	0.159	2.92E-14	3.62E-10	1
IL4	2.433	0.806	0.375	0	0	2
CD40LG	0.873	0.984	0.81	2.20E-245	2.74E-241	2
XCL1	2.095	0.908	0.736	2.78E-238	3.45E-234	2
IFNG	1.020	1	0.999	1.88E-197	2.33E-193	2
XCL2	1.883	0.878	0.697	1.18E-188	1.47E-184	2

IL13	1.815	0.325	0.1	1.66E-105	2.06E-101	2
LINC00152	0.576	0.877	0.69	1.36E-77	1.69E-73	2
IL10	1.077	0.174	0.039	3.31E-70	4.12E-66	2
RRAGA	0.688	0.522	0.28	1.48E-64	1.84E-60	2
MIR4435-1HG	0.745	0.372	0.16	3.30E-62	4.10E-58	2
MIR155HG	0.511	0.84	0.644	4.55E-62	5.65E-58	2
CSF2	0.849	0.826	0.682	1.94E-60	2.42E-56	2
EVI2A	0.464	0.847	0.664	6.35E-58	7.90E-54	2
H3F3B	0.376	0.998	0.997	9.14E-58	1.14E-53	2
AC013264.2	0.804	0.4	0.195	9.43E-56	1.17E-51	2
ADAM19	0.598	0.381	0.179	4.70E-52	5.84E-48	2
CFLAR	0.538	0.688	0.486	3.62E-49	4.50E-45	2
TNFSF10	0.477	0.107	0.027	6.47E-38	8.04E-34	2
FGL2	0.673	0.161	0.056	6.62E-37	8.23E-33	2
RTKN2	0.476	0.151	0.049	8.40E-37	1.04E-32	2
TNFRSF4	0.782	0.271	0.131	1.43E-34	1.78E-30	2
IL2	1.975	0.782	0.72	3.53E-33	4.38E-29	2
PPP1R2	0.464	0.466	0.301	3.39E-30	4.22E-26	2
PHLDA2	0.477	0.133	0.048	3.43E-28	4.26E-24	2
GADD45G	0.490	0.156	0.067	2.84E-24	3.53E-20	2
MAP3K8	0.410	0.386	0.245	1.58E-23	1.97E-19	2
DUSP4	0.419	0.172	0.077	1.58E-22	1.96E-18	2
NAMPT	0.397	0.401	0.282	3.45E-17	4.28E-13	2
TNFSF8	0.376	0.125	0.061	2.58E-14	3.21E-10	2
AREG	0.367	0.367	0.28	5.49E-10	6.83E-06	2
GZMH	1.197	0.287	0.017	1.30E-171	1.62E-167	3
KLRD1	1.903	0.713	0.159	1.94E-160	2.41E-156	3
GNLY	1.704	0.904	0.394	2.13E-134	2.65E-130	3
GZMB	2.815	0.86	0.368	9.85E-125	1.22E-120	3
CLECL1	1.205	0.693	0.22	1.32E-83	1.64E-79	3
MIR4435-1HG	1.349	0.594	0.172	2.16E-81	2.68E-77	3
ZEB2	0.919	0.283	0.055	5.12E-57	6.37E-53	3
YBX3	0.676	0.15	0.02	9.25E-45	1.15E-40	3
KLRC1	1.273	0.222	0.044	2.92E-43	3.63E-39	3
NKG7	0.750	0.901	0.624	1.66E-42	2.06E-38	3
CCL4	0.480	1	1	6.62E-38	8.22E-34	3
XCL2	1.011	0.867	0.716	4.71E-34	5.85E-30	3
LINC00152	0.571	0.942	0.706	6.66E-34	8.27E-30	3
GAPDH	0.432	0.98	0.899	1.18E-26	1.47E-22	3
XCL1	0.551	0.884	0.754	7.22E-25	8.98E-21	3
CCL4L1	0.463	0.904	0.647	1.57E-23	1.95E-19	3

HLA-DRB5	0.428	0.113	0.022	1.89E-21	2.35E-17	3
CD226	0.529	0.184	0.067	1.96E-14	2.44E-10	3
HLA-DRB1	0.440	0.119	0.034	4.10E-14	5.10E-10	3
MAP2K3	0.446	0.3	0.145	6.89E-13	8.56E-09	3
TNFRSF9	0.575	0.341	0.184	2.48E-12	3.08E-08	3
LYST	0.425	0.686	0.511	1.16E-11	1.44E-07	3
VMP1	0.431	0.696	0.497	2.15E-11	2.67E-07	3
CD72	0.416	0.143	0.054	7.86E-11	9.77E-07	3
LAG3	0.393	0.14	0.053	2.52E-10	3.13E-06	3
CBLB	0.437	0.324	0.182	3.84E-10	4.77E-06	3
HOPX	0.423	0.263	0.136	1.22E-09	1.52E-05	3
IL13	0.438	0.232	0.126	1.79E-07	0.00222412	3
GABARAPL1	0.426	0.188	0.097	2.35E-07	0.00291448	3
LGALS1	0.415	0.229	0.129	7.97E-07	0.00990507	3
CD200	1.511	0.383	0.023	7.87E-224	9.77E-220	4
GEM	2.083	0.614	0.122	1.02E-139	1.26E-135	4
LYPD3	0.733	0.148	0.005	4.88E-119	6.06E-115	4
SELL	0.828	0.188	0.011	5.48E-111	6.81E-107	4
PKIA	0.894	0.22	0.021	2.31E-88	2.87E-84	4
ICOS	1.136	0.422	0.081	1.94E-85	2.41E-81	4
YBX3	0.973	0.199	0.019	2.97E-80	3.69E-76	4
PGAP1	1.096	0.271	0.036	6.10E-79	7.58E-75	4
FDX1	1.588	0.502	0.144	5.08E-68	6.31E-64	4
NIN	0.824	0.209	0.027	6.99E-63	8.68E-59	4
CD55	1.224	0.708	0.32	2.17E-62	2.70E-58	4
THAP2	1.259	0.567	0.204	5.74E-58	7.13E-54	4
CD69	0.912	0.996	0.98	8.80E-55	1.09E-50	4
BCL2A1	0.783	0.953	0.781	1.60E-54	1.99E-50	4
MT2A	1.617	0.657	0.312	1.19E-51	1.48E-47	4
MYC	0.790	0.949	0.753	6.71E-48	8.34E-44	4
RGS10	0.953	0.646	0.291	1.14E-47	1.42E-43	4
MIR155HG	0.758	0.913	0.662	2.95E-45	3.67E-41	4
CYTIP	0.986	0.668	0.323	4.29E-44	5.33E-40	4
IL2RA	1.019	0.357	0.099	4.75E-44	5.90E-40	4
IRF1	0.870	0.314	0.086	2.20E-38	2.74E-34	4
PELI1	0.796	0.235	0.057	1.81E-33	2.26E-29	4
PHF6	0.765	0.242	0.061	1.09E-32	1.36E-28	4
FOXP1	0.812	0.556	0.254	3.01E-32	3.74E-28	4
SOD1	0.919	0.643	0.369	6.68E-29	8.30E-25	4
AREG	0.833	0.556	0.282	2.35E-28	2.92E-24	4
RASA1	0.761	0.112	0.018	3.11E-27	3.86E-23	4

ITM2A	0.820	0.523	0.252	6.42E-27	7.98E-23	4
SOCS3	0.736	0.628	0.355	7.21E-27	8.96E-23	4
CLEC2D	0.843	0.513	0.251	1.13E-25	1.40E-21	4
TXNIP	1.619	0.293	0.014	4.29E-163	5.34E-159	5
TSC22D3	1.957	0.754	0.171	1.08E-121	1.34E-117	5
DUSP1	2.057	0.602	0.139	8.10E-91	1.01E-86	5
DNAJB1	2.201	0.461	0.088	1.79E-76	2.22E-72	5
HSPA1B	1.869	0.162	0.011	1.97E-67	2.45E-63	5
FOS	1.926	0.806	0.45	4.59E-59	5.70E-55	5
IL32	1.110	0.901	0.627	6.40E-51	7.96E-47	5
HSPA1A	1.740	0.131	0.011	3.72E-46	4.62E-42	5
JUN	1.669	0.602	0.25	1.92E-44	2.38E-40	5
PFN1	1.092	0.843	0.548	6.55E-44	8.14E-40	5
TNFAIP3	1.463	0.634	0.291	2.99E-42	3.71E-38	5
GIMAP4	0.928	0.188	0.029	1.55E-35	1.93E-31	5
RP4-539M6.22	1.435	0.325	0.087	5.31E-33	6.60E-29	5
ANAPC16	1.053	0.356	0.104	4.05E-32	5.03E-28	5
NFKBIA	1.297	0.791	0.59	9.42E-31	1.17E-26	5
MYL12A	0.925	0.77	0.542	1.65E-30	2.05E-26	5
TRAF3IP3	0.871	0.157	0.024	3.06E-30	3.80E-26	5
PTPRCAP	1.069	0.581	0.293	8.58E-29	1.07E-24	5
EGR1	0.932	0.634	0.374	2.75E-25	3.42E-21	5
ARL5B	1.109	0.377	0.139	4.10E-25	5.09E-21	5
PPP1R15A	1.143	0.445	0.2	9.57E-25	1.19E-20	5
LTB	1.050	0.691	0.463	9.93E-24	1.23E-19	5
BIN2	0.880	0.225	0.059	2.90E-22	3.60E-18	5
LYAR	0.999	0.482	0.238	8.20E-22	1.02E-17	5
CKLF	0.953	0.277	0.091	5.70E-20	7.08E-16	5
MIR24-2	1.243	0.366	0.152	1.26E-19	1.56E-15	5
PYURF	0.877	0.173	0.041	3.78E-19	4.70E-15	5
FOSB	0.927	0.403	0.198	4.12E-16	5.12E-12	5
CD69	0.871	0.953	0.981	1.42E-15	1.76E-11	5
HSP90AA1	0.985	0.576	0.473	1.38E-09	1.72E-05	5

Supplementary Table 3. The subset discriminative genes in Cluster2 of stimulated human NKT cells

Gene	log(Fold-Change)	% Cells inside Cluster	% Cells outside Cluster	p-value	Adjusted p-value	Cluster
CCL5	1.015	0.982	0.903	6.61E-26	8.22E-22	1
XCL2	1.649	0.904	0.641	3.32E-25	4.12E-21	1
IL4	1.819	0.845	0.456	4.10E-25	5.10E-21	1
XCL1	1.323	0.93	0.709	6.87E-24	8.54E-20	1
IFNG	0.993	1	1	1.35E-23	1.67E-19	1
CCL4	1.257	0.999	1	7.08E-21	8.80E-17	1
CCL4L2	1.783	0.681	0.243	1.37E-19	1.71E-15	1
CCL4L1	1.531	0.581	0.194	3.90E-14	4.85E-10	1
CCL3L3	1.742	0.53	0.175	1.44E-12	1.79E-08	1
IL13	2.187	0.355	0.049	2.60E-10	3.23E-06	1
KLRB1	0.511	0.867	0.641	1.11E-09	1.38E-05	1
GZMK	0.668	0.589	0.282	3.33E-08	0.0004	1
RAB27A	0.732	0.513	0.233	1.10E-07	0.0014	1
CCL3	1.068	0.961	0.922	3.00E-07	0.0037	1
LINC00152	0.330	0.897	0.699	5.89E-07	0.0073	1
IRF8	0.756	0.365	0.136	4.14E-06	0.0515	1
CCL3L1	2.015	0.499	0.33	1.34E-05	0.1671	1
MAP3K8	0.631	0.409	0.184	2.69E-05	0.3346	1
NFKBIA	0.690	0.577	0.417	7.14E-05	0.8878	1
HSP90AA1	0.431	0.526	0.301	0.0001	1	1
POLR2K	0.372	0.781	0.602	0.0001	1	1
IRF4	0.590	0.115	0	0.0003	1	1
MIR4435-1HG	0.603	0.39	0.204	0.0003	1	1
PLEK	0.595	0.323	0.155	0.0004	1	1
GADD45B	0.383	0.8	0.689	0.0004	1	1
LYST	0.386	0.563	0.379	0.0005	1	1
GCLM	0.383	0.363	0.175	0.0006	1	1
CLECL1	0.521	0.297	0.136	0.0007	1	1
CD7	0.294	0.584	0.398	0.0007	1	1
CDC42SE1	0.655	0.193	0.058	0.0008	1	1
IL18RAP	0.580	0.178	0.049	0.0008	1	1
NKG7	0.535	0.476	0.33	0.0009	1	1
RRAGA	0.307	0.536	0.388	0.0018	1	1
SEMA7A	0.473	0.148	0.039	0.0023	1	1

RASGEF1B	0.534	0.22	0.097	0.0032	1	1
CBLB	0.453	0.246	0.117	0.0034	1	1
SERPINB9	0.373	0.556	0.388	0.0037	1	1
IFNGR1	0.430	0.156	0.049	0.0042	1	1
PSMC4	0.388	0.137	0.039	0.0055	1	1
RNF19A	0.330	0.551	0.379	0.0057	1	1
DUSP1	0.408	0.16	0.058	0.0066	1	1
ARFGAP3	0.404	0.118	0.029	0.0067	1	1
CD164	0.346	0.38	0.243	0.0070	1	1
PHLDA2	0.541	0.143	0.049	0.0071	1	1
MAFF	0.418	0.197	0.087	0.0080	1	1
ARHGEF3	0.390	0.251	0.136	0.0083	1	1
TYROBP	0.301	0.146	0.049	0.0089	1	1
RUNX1	0.440	0.127	0.039	0.0092	1	1
FOS	0.439	0.548	0.437	0.0096	1	1
DEK	0.389	0.228	0.117	0.0098	1	1
IL2	1.946	0.99	0.76	9.30E-33	1.16E-28	2
LPAR6	1.079	0.398	0.084	1.72E-22	2.13E-18	2
AC020571.3	1.322	0.282	0.043	9.17E-22	1.14E-17	2
TMSB4X	0.594	1	1	5.14E-17	6.38E-13	2
RGCC	0.533	1	0.998	6.82E-17	8.47E-13	2
ITGB1	1.099	0.476	0.162	1.53E-16	1.90E-12	2
B2M	0.260	1	1	9.02E-16	1.12E-11	2
IL10	1.625	0.437	0.145	9.92E-16	1.23E-11	2
TNFRSF4	1.390	0.534	0.243	5.46E-14	6.78E-10	2
ICOS	0.763	0.388	0.123	3.56E-13	4.42E-09	2
IL7R	0.425	0.951	0.872	3.82E-11	4.74E-07	2
S100A4	0.508	0.874	0.706	6.12E-11	7.60E-07	2
TMSB10	0.442	0.951	0.828	2.09E-09	2.59E-05	2
FTH1	0.382	1	1	3.10E-09	3.86E-05	2
CD52	0.487	0.845	0.615	3.42E-09	4.25E-05	2
TNFSF8	0.858	0.301	0.105	3.52E-09	4.37E-05	2
REXO4	0.480	0.117	0.018	7.40E-09	9.20E-05	2
H3F3B	0.466	1	0.998	1.88E-08	0.0002	2
RP11-403A21.2	0.744	0.32	0.124	3.26E-08	0.0004	2
EZR	0.411	0.922	0.852	9.87E-08	0.0012	2
DDIT4	0.586	0.689	0.466	1.95E-07	0.0024	2
LGALS1	0.602	0.33	0.151	1.01E-06	0.0125	2
SUB1	0.340	0.913	0.807	1.05E-06	0.0130	2
GLUD1	0.409	0.602	0.369	3.44E-06	0.0427	2

ACTB	0.420	0.903	0.829	3.82E-06	0.0475	2
TPT1	0.261	0.99	0.961	8.75E-06	0.1088	2
CLEC2D	0.547	0.447	0.262	1.07E-05	0.1334	2
VMP1	0.329	0.767	0.604	1.41E-05	0.1753	2
VIM	0.285	0.951	0.896	2.75E-05	0.3423	2
PDP1	0.399	0.146	0.046	2.99E-05	0.3713	2
FOXP1	0.469	0.456	0.274	3.03E-05	0.3763	2
NDUFV2	0.467	0.291	0.139	3.07E-05	0.3813	2
HMCES	0.531	0.223	0.094	3.62E-05	0.4499	2
FDX1	0.909	0.262	0.133	4.35E-05	0.5405	2
GLIPR1	0.605	0.524	0.369	5.57E-05	0.6919	2
GEM	0.883	0.194	0.082	7.66E-05	0.9522	2
RTKN2	0.546	0.282	0.137	7.68E-05	0.9544	2
TPM4	0.582	0.272	0.137	7.75E-05	0.9630	2
PLAUR	0.800	0.262	0.132	8.58E-05	1	2
GPR171	0.514	0.65	0.504	8.59E-05	1	2
CD200	0.434	0.136	0.045	8.81E-05	1	2
ACTR3	0.479	0.34	0.186	9.02E-05	1	2
YWHAQ	0.399	0.583	0.421	0.0001	1	2
RP11-347P5.1	0.404	0.65	0.484	0.0001	1	2
ITM2A	0.592	0.476	0.317	0.0001	1	2
MYL12A	0.395	0.563	0.406	0.0001	1	2
SFXN1	0.551	0.126	0.043	0.0002	1	2
NR3C1	0.618	0.35	0.206	0.0002	1	2
HLA-DPB1	0.538	0.359	0.217	0.0002	1	2
NSMCE4A	0.363	0.107	0.033	0.0003	1	2
S100A6	0.369	0.602	0.451	0.0003	1	2
UCP2	0.462	0.427	0.28	0.0003	1	2
ARID5B	0.355	0.893	0.772	0.0003	1	2
INSIG1	0.525	0.515	0.386	0.0003	1	2
ANKRD12	0.504	0.194	0.087	0.0003	1	2
CD2	0.494	0.495	0.372	0.0003	1	2
LPXN	0.482	0.282	0.153	0.0004	1	2
LMNA	0.386	0.825	0.777	0.0005	1	2
TRAPPC1	0.355	0.117	0.04	0.0005	1	2
ELF1	0.376	0.534	0.371	0.0005	1	2
ACTG1	0.666	0.476	0.363	0.0006	1	2
LTB	0.442	0.505	0.388	0.0006	1	2
LRRC8C	0.384	0.184	0.082	0.0006	1	2
TRIM69	0.383	0.117	0.041	0.0007	1	2
TNFSF10	0.423	0.204	0.097	0.0007	1	2

ANXA1	0.292	0.981	0.971	0.0008	1	2
MGAT4A	0.350	0.117	0.041	0.0008	1	2
TAP1	0.395	0.107	0.037	0.0008	1	2
STK24	0.421	0.117	0.043	0.0008	1	2
SPRY1	0.426	0.107	0.037	0.0009	1	2
ITM2B	0.440	0.563	0.429	0.0010	1	2
MYCBP2	0.284	0.126	0.047	0.0010	1	2
NUDT4	0.362	0.282	0.162	0.0011	1	2
GRWD1	0.311	0.117	0.043	0.0011	1	2
EMP1	0.359	0.175	0.08	0.0013	1	2
SOCS1	0.423	0.534	0.404	0.0013	1	2
VAPA	0.341	0.476	0.338	0.0014	1	2
IL2RA	0.364	0.223	0.115	0.0014	1	2
HIGD2A	0.376	0.272	0.157	0.0015	1	2
AREG	0.644	0.485	0.354	0.0017	1	2
CD82	0.408	0.214	0.112	0.0019	1	2
ALOX5AP	0.456	0.194	0.101	0.0023	1	2
ATP1A1	0.293	0.485	0.352	0.0025	1	2
TMEM123	0.292	0.214	0.11	0.0025	1	2
ANKRD28	0.520	0.155	0.074	0.0027	1	2
PCBP2	0.371	0.398	0.278	0.0030	1	2
SAMSN1	0.380	0.612	0.534	0.0031	1	2
KLF6	0.266	0.757	0.659	0.0031	1	2
USP53	0.331	0.136	0.059	0.0032	1	2
SOD1	0.368	0.515	0.406	0.0033	1	2
HSPH1	0.353	0.32	0.209	0.0034	1	2
CDCA4	0.317	0.107	0.043	0.0035	1	2
ATP5G2	0.339	0.427	0.307	0.0036	1	2
TCEB2	0.297	0.262	0.153	0.0037	1	2
PNP	0.330	0.214	0.118	0.0041	1	2
CNOT6L	0.391	0.301	0.189	0.0047	1	2
RPS17L	0.454	0.117	0.051	0.0049	1	2
ODC1	0.379	0.223	0.13	0.0053	1	2
CSNK1A1	0.261	0.33	0.215	0.0056	1	2
TGFBR2	0.393	0.184	0.102	0.0057	1	2
NME1-NME2	0.290	0.146	0.069	0.0058	1	2
TSTD1	0.387	0.175	0.091	0.0058	1	2
NSMCE1	0.399	0.117	0.052	0.0060	1	2
SOCS3	0.549	0.466	0.368	0.0060	1	2
AC006369.2	0.389	0.184	0.099	0.0063	1	2
SNRPG	0.294	0.641	0.543	0.0063	1	2

AHR1	0.291	0.117	0.051	0.0064	1	2
LAPTM5	0.275	0.33	0.218	0.0066	1	2
C4orf48	0.279	0.233	0.136	0.0066	1	2
TANK	0.341	0.165	0.085	0.0078	1	2
CORO1A	0.419	0.33	0.229	0.0080	1	2
NSRP1	0.293	0.136	0.066	0.0085	1	2
NR1H2	0.334	0.155	0.082	0.0088	1	2
BIRC3	0.312	0.757	0.746	0.0092	1	2

Supplementary Table 4. Summary of features of human NKT cell clusters

<table>
<tr><th>Treatment</th><th>Cluster Name</th><th colspan="2">Functional Features</th></tr>
<tr><td rowspan="4">Unstimulated</td><td>UnstimC1</td><td colspan="2">nonspecific</td></tr>
<tr><td>UnstimC2</td><td colspan="2">enhanced motility and tissue resident capability</td></tr>
<tr><td>UnstimC3</td><td colspan="2">enhanced cytotoxic function</td></tr>
<tr><td>UnstimC4</td><td colspan="2">relative naïve, immature with advanced expansion and homeostasis capacity</td></tr>
<tr><td rowspan="6">Stimulated</td><td>StimC1</td><td colspan="2">enhanced multiple chemokine expression, with sparsely NKT17 distribution</td></tr>
<tr><td rowspan="2">StimC2</td><td>StimC2-A</td><td>upregulated XCLs, IFNγ, IL4, IL13 and CCL chemokines</td></tr>
<tr><td>StimC2-B</td><td>upregulated IL2, IL10, ICOS, OX40 with putative immune regulatory function.</td></tr>
<tr><td>StimC3</td><td colspan="2">enhanced cytotoxic function</td></tr>
<tr><td>StimC4</td><td colspan="2">relative immature with enhance cell growth and survival capacity</td></tr>
<tr><td>StimC5</td><td colspan="2">representatives of early or transitional stage of exhaustion or anergy</td></tr>
</table>
